# Prognostic significance of cyclooxygenase-2 (COX-2) expression in patients with surgically resectable adenocarcinoma of the oesophagus

**DOI:** 10.1186/1471-2407-6-134

**Published:** 2006-05-19

**Authors:** Pradeep Bhandari, Adrian C Bateman, Raj L Mehta, Bernard SF Stacey, Penny Johnson, Ian A Cree, Federica Di Nicolantonio, Praful Patel

**Affiliations:** 1 Department of Gastroenterology, Southampton University Hospital, Southampton, SO166YD, UK; 2 Department of Cellular Pathology, Southampton University Hospital, Southampton, SO166YD, UK; 3 Medical Statistics, Southampton University Hospital, Southampton, SO166YD, UK; 4 Translational Oncology Research Centre, Queen Alexandra Hospital, Portsmouth. PO6 3LY, UK

## Abstract

**Background:**

COX-2 expression in tumour cells has been associated with poor prognosis in gastrointestinal and non-gastrointestinal cancers. The aim of our study was to test the hypothesis that higher levels of COX-2 expression are prognostically related to poor clinico-pathologic features in adenocarcinoma of the oesophagus.

**Methods:**

We reviewed the records of 100 consecutive patients undergoing resection for adenocarcinoma of the oesophagus to collect data on T-stage, N-stage, tumour recurrence and survival. T & N-stage was further confirmed by histological examination. COX-2 protein expression was assessed by immunohistochemistry in all patients and COX-2 m-RNA expression was measured by quantitative RT-PCR in a small group of patients.

**Results:**

Higher levels of COX-2 expression were associated with higher T stage (p = 0.008), higher N stage (p = 0.049), increased risk of tumour recurrence (p = 0.01) and poor survival (p = <0.001). A COX-2 score of >200 was associated with a median survival of 10 months compared to 26 months with a score of <200 (p = <0.001).

**Conclusion:**

Higher levels of COX-2 expression are associated with poor clinico-pathologic features and poor survival in patients with oesophageal adenocarcinoma.

## Background

Oesophageal adenocarcinoma is an aggressive human malignancy. It is of particular concern that the incidence of oesophageal adenocarcinoma is increasing more rapidly then any other cancer in Western Europe and the United States[1]. It has an incidence of 7.4 per 100,000 in England and Wales and accounts for 5,600 deaths annually [2,3]. The only hope of cure is complete surgical resection with or without neo-adjuvant chemotherapy. Endoscopic ultrasound has improved the accuracy of local staging leading to better patient selection for curative resection and estimating prognosis. Unfortunately, the 5-year survival rate is only 25% even after intentionally curative resection with a median survival of 15–18 months[4]. Developing better prognostic markers and effective treatment strategies depends on our understanding of the biological behaviour of these tumours and identifying factors responsible for tumour invasiveness, metastasis and recurrence.

Epidemiologic studies show that non-steroidal anti-inflammatory drugs (NSAIDs) can reduce the incidence and mortality from gastrointestinal malignancy, including oesophageal, gastric and colonic cancers[5]. This suggests a pathogenic role of cyclooxygenase (COX), the principle target enzyme for the actions of NSAIDs, in gastrointestinal tumourigenesis. COX is a rate-limiting enzyme involved in the conversion of arachidonic acid to prostaglandins, prostacyclin, and thromboxanes. Two COX genes, COX-1 and COX-2 have been identified, which share over 60% identity at the amino acid level[6]. COX-1 is constitutively expressed in many tissues and responsible for various physiological functions[7]. COX-2 is not universally expressed by all tissues but can be induced by various pathologic stimuli, such as inflammation, cytokines and various growth factors produced by tumour cells [8-10]. Increased expression of COX-2 has been demonstrated in various inflammatory diseases[11] and malignancies of the colon[12] stomach[13], liver[14], pancreas[15] and oesophagus[16]. Overexpression of COX-2 in human carcinomas seems to be of functional significance as double knockout mice for APC and COX-2 genes showed marked reduction in the size and frequency of intestinal polyps[17]. This anti-neoplastic effect of COX-2 inhibitors was further confirmed when they were found to reduce polyp burden in patients with familial adenomatous polyposis[18]. There is also cumulative evidence that selective COX-2 inhibitors prevent carcinogenesis in experimental animals[19], and that these compounds induce apoptosis[20], inhibit angiogenesis[21] and growth[22] in several types of cancer cells. These findings suggest that COX-2 may be associated with carcinogenesis and progression of certain types of human malignancies. However, the prognostic significance of COX-2 expression in human oesophageal carcinoma remains unclear. The clinicopathologic significance of COX-2 in oesophageal carcinoma has been reported recently with conflicting results [23-26]. We have further evaluated the role of COX-2 in patients with adenocarcinoma of oesophagus. The aim of our study was to test the hypothesis that higher levels of COX-2 expression are related to higher pathological tumour stage and poor clinical outcome in adenocarcinoma of oesophagus.

## Methods

### Patient population

We reviewed the records of 100 consecutive patients with adenocarcinoma of oesophagus that underwent oesophageal resection with curative intent at our institute between 1990–96. The data was collected in 2001 to ensure a minimum follow-up of 5 years (range: 5–11 yr). None of these patients received preoperative chemotherapy or irradiation. Preoperative work up included gastroscopy with biopsy, abdominal ultrasound, chest X-ray and CT scan (selected cases) of chest and abdomen. All these patients underwent Ivor lewis oesophagectomy. We excluded patients with squamous cell cancer of oesophagus, adenocarcinomas crossing the gastroesophageal junction and those with distant metastasis. All pathology reports and archival material from these patients were reviewed by one of the investigators (ACB) to ascertain the T-stage, N-stage and to identify the block with deepest invasion of tumour. Recurrence and survival data were obtained from the case notes as these patients were followed up regularly in the outpatient clinics.

We prospectively collected fresh tissue biopsies from a separate group (not a part of the 100 consecutive patiets) of sixteen consecutive patients with oesophageal adenocarcinoma to compare COX-2 m-RNA expression, measured by quantitative RT-PCR with COX-2 protein expression, measured by immunohistochemistry.

The study was approved by the Southampton and South West Hants Local Research Ethics Committee. (ref:292/01)

### Immunohistochemistry

The resection specimens were fixed in 10% buffered formalin and embedded in paraffin wax. Four micron sections were cut from each block and mounted onto glass slides before being de-paraffinised and then immersed in 0.3% H_2_O_2 _in methanol for 10 minutes to block endogenous peroxidase activity. The sections were pressure cooked for 12 minutes in citrate buffer (pH 6.1) for antigen retrieval. Visualisation of the bound primary antibodies was performed using AEC as a chromogen.

Antigen adsorption studies were performed using COX-2 blocking peptide. The sections were treated in the same manner as above except that COX-2 antibody was preincubated with 10 mcg/ml COX-2 blocking peptide at room temperature for one hour. Positive controls (tonsil) were used for each run and a negative control (primary antibody replaced by TBS) was included for each section. Scoring was performed using an intensity-proportion scoring system, as previously described for immuno-scoring of oestrogen receptors in breast carcinoma[27] and recently adapted by us for immuno-scoring in upper gastrointestinal biopsies[28]. The percentage of positively labelled cells (0–100%) in each section was multiplied by the intensity of labelling, graded from 0 (no labelling) to 3 (intense labelling), providing a score between 0 and 300. In adenocarcinoma of oesophagus, it is not known if intensity of staining is more significant then the proportion of cells staining positive or vice versa so we decided to use this system that can take account of both the intensity and proportion of positively staining cells.

The primary antibody to COX-2 (Cat. No. 160112) and the blocking peptide for COX-2 (Cat.No. 360107) were sourced from Cayman Chemical Company (Ann Arbor, Michigan, USA).

### Reproducibility and scoring validation

During our initial experiments, we stained a group of 10 specimens on three separate occasions to ascertain the reproducibility of the COX-2 staining. After a time in excess of 3 months, 10 sections were selected at random and rescored to assess the intraobserver variation in scoring. This was carried out by the original investigator without prior knowledge of the clinicopathologic characteristics of the tumour or the previous COX-2 score.

### Quantitative RT-PCR

Tumor cells were obtained after enzymatic dissociation from endoscopic oesophageal biopsies and were resuspended in RNA later (Ambion). Cells were stored at -80°C until total RNA was extracted with a commercially available kit (NucleoSpin^® ^RNA II mini, Macherey-Nagel, Germany, Cat. 740955) following manufacturer's instructions. Total RNA was then reverse-transcribed by using the Promega reverse transcription system (Promega, Southampton, UK, Cat # A3500). The resulting c-DNA was amplified by real-time quantitative PCR on a Biorad iCycler instrument (BioRad Laboratories, Hemel Hampstead, UK) using SYBR Green PCR buffer (SYBR Green PCR Core Reagents, Applied Biosystems, P/N 4304886). The following primers (400 nM) were used for COX-2 detection (forward 5'-CCTTCCTCCTGTGCCTGATG; reverse 5'-ACAATCTCATTTGAATCAGGAAGCT), as previously described (Sales et al., 2002). It is well known that mRNA levels of commonly used house keeping genes vary between individuals and the use of a single housekeeping gene within a qRT-PCR system is not sufficient. (Vandesompele et al., 2002) At least three out of the following four housekeeping genes were used for each experiment: glyceraldehyde-3 phosphate dehydrogenase (GAPDH), hypoxanthine phosphoribosyltransferase 1 (HPRT1), human porphobilinogen deaminase (PBGD) and TATA box binding protein (TBP). A positive control (pooled c-DNA from a variety of human tumors, including breast, ovarian, colorectal and oesophageal carcinoma) and negative controls with no template and RT-negative as template were added in every experiment. All assays were run in triplicate. A comparative Ct (cycle threshold) method was employed to measure relative gene expression (ABI PRISM 7700 User Bulletin #2, 2001 update). The following formula was used to calculate the relative amount of the transcript in the sample: 2^-ΔCt^, where ΔCt is the difference in Ct between the gene of interest (COX-2) and the mean of the two most stable and therefore most appropriate house keeping/reference genes.

### Immunostaining vs RT-PCR

In 16 patients prospective biopsies were used to assess the COX-2 protein expression by immuno-staining and COX-2 m-RNA by RT-PCR. These values were then compared to assess the relationship between COX-2 m-RNA and protein expression.

### Statistics

Statistical analysis was performed using SPSS 11.0 software. The association between demographic, clinicopathologic features and COX-2 was analysed using the student t test (continuous data) and chi square test (categorical data). Analysis of variance was performed and Fishers exact test was performed whenever necessary. The Cox proportional hazards model was used to examine the effect of COX-2 expression in relation to other prognostic factors. A p-value of 0.05 or less was considered to be statistically significant.

## Results

### Clinical data

Out of the 100 consecutive patients who underwent potentially curative surgery for adenocarcinoma of oesophagus, 6 patients died within 1 month (overall 30 day mortality of 6%) and 4 patients did not have clear (cancer free) resection margins so were excluded from survival analysis. We therefore performed the survival analysis on the data obtained from 90 patients (M: 81, F: 9) with a mean age of 65 yrs (39–83 yrs). The data on Tumour and Nodal stage of the tumours is shown in table-2. In 16 patients, we collected prospective biopsies and compared COX-2 m-RNA expression (RT-PCR) with COX-2 protein expression.

### COX-2 expression

**Figure 1:**
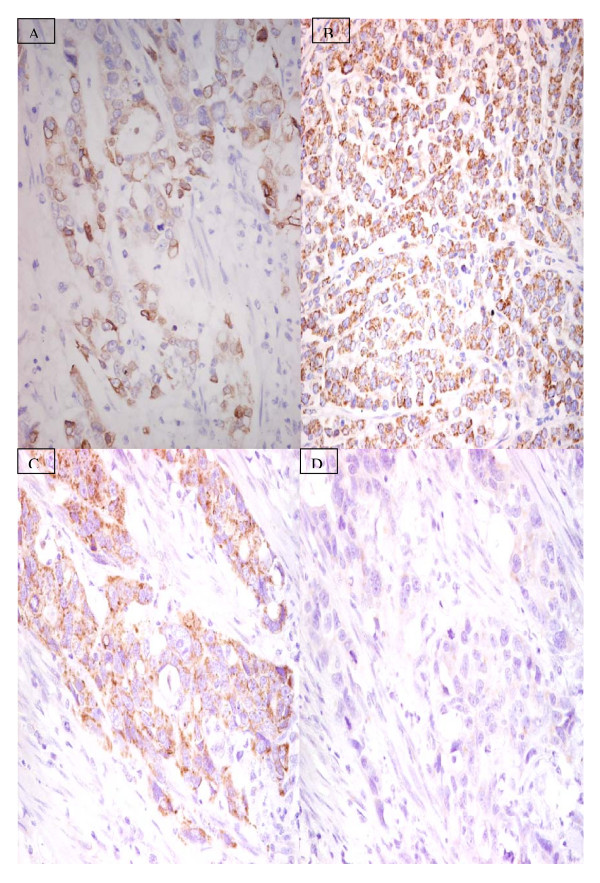
Representative examples of COX-2 immunhistochemistry in adenocarcinoma of oesophagus. (A) low level (<200) and (B) high level (>200)of COX-2 expression. Antigen adsorption study demonstrated good immunoreactivity with COX-2 antibody (C) and lack of immunoreactivity when staining was repeated in the presence of COX-2 blocking peptide (D).

COX-2 expression was seen in all patients (100%) and was primarily expressed by tumour cells. The degree of expression was variable but the median COX-2 score was 200 and we used this as a threshold value with fig-1(A) demonstrating low (<200) and fig1(B) demonstrating high (>200) levels of expression. The antigen adsorption study demonstrated the specificity of our antibody by showing good immunoreactivity (fig-1(C)) with COX-2 antibody and complete blockage of this immunoreactivity (fig-1(D)) when staining was repeated in the same specimen with COX-2 antibody preincubated with COX-2 blocking peptide. The specificity of our antibody was further confirmed by the lack of staining seen in our negative controls (primary antibody replaced by TBS).

### Reproducibility and scoring validation

The 10 specimen that were stained on three separate occasions showed a very good reproducibility for COX-2 staining. Of the 10 sections being rescored, we found that intensity score was correctly reproduced in all 10 and the proportion score was correctly reproduced in 7 sections. The remaining 3 sections, which were incorrectly scored, were all from the low COX-2 expressing group but the discrepancies were small, giving an average error of <5%.

### Correlation between COX-2 protein and COX-2 m-RNA expression

**Figure 2:**
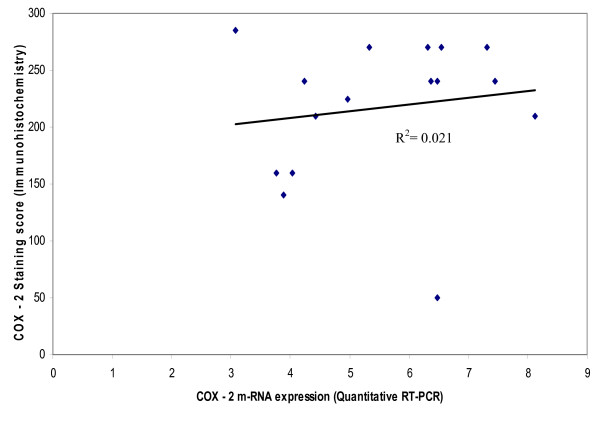
Correlation between COX-2 m-RNA and COX-2 protein expression in biopsies from oesophageal adenocarcinoma.

Fig-2 shows a positive correlation (R^2 ^= 0.021) between COX-2 m-RNA expression, assessed by RT-PCR and COX-2 protein expression, assessed by immunohistochemistry in oesophageal biopsies from patients with adenocarcinoma of oesophagus.

### Correlation between survival and clinico-pathologic parameters

**Figure 3:**
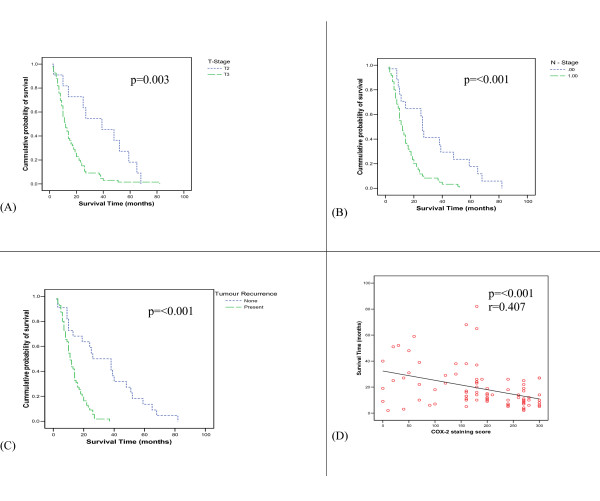
Correlation between survival and clinicopathologic parameters: Kaplan-Meier curves comparing survival with tumour invasion (A), nodal metastasis (B) and tumour recurrence (C). Linear regression (D) analysis showing negative correlation between survival and COX-2 expression.

**Figure 4:**
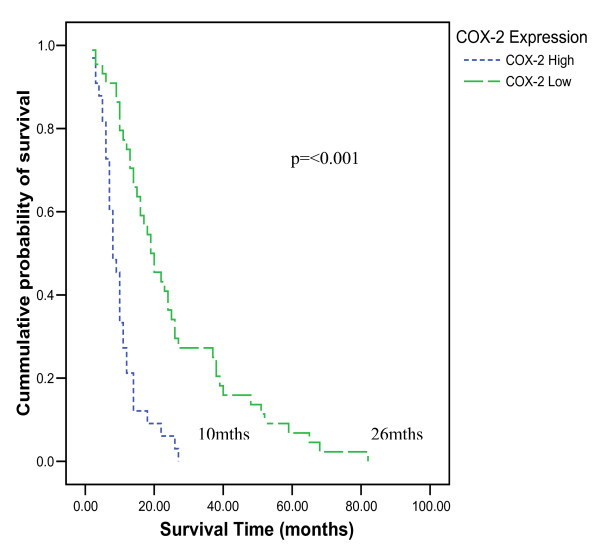
Kaplan-Meier survival curves according to COX-2 expression illustrates a poor survival (median survival of 10 mths) in patients with tumours expressing High (≥200) levels of COX-2 and better survival (median survival of 26 mths) in those with Low (<200) levels of COX-2.

Fig-3 shows the relationship between survival and clinico-pathologic factors. We confirmed a significant correlation between survival and traditional prognostic factors including the depth of tumour invasion or T-stage (fig 3-A), nodal metastasis or N-stage (fig 3-B) and tumour recurrence (fig 3-C). We also demonstrated (fig 3-D) a significant negative correlation between patient survival and COX-2 expression in the oesophageal biopsies taken from the tumour sites (p = <0.001). On further analysis we identified that using our scoring system a COX-2 expression score of (≥200) served as a good marker of poor survival, with a median survival time of 10 months as compared to 26 months in patients with COX-2 expression <200 (p = <0.001) as shown in fig-4.

We performed univariate and multivariate survival analysis using Cox proportional Hazards model and confirmed that higher T-stage, N-stage and COX-2 score were independent markers of survival (table-1)

### Correlation between clinicopathologic characteristics and COX-2 expression

Table-2 illustrates that higher levels of COX-2 expression were associated with higher T-stage (p = 0.008), higher N-stage (p = 0.049), poorly differentiated tumour on histology (p = 0.045) and higher incidence of tumour recurrence (p = 0.01) after potentially curative resection of adenocarcinoma of oesophagus.

## Discussion

Our results demonstrate that the COX-2 enzyme is universally expressed in adenocarcinoma of oesophagus but the amount of COX-2 expression is variable. Tumours expressing high levels of COX-2 are associated with poor patient survival after surgery. We also demonstrated a relationship between COX-2 expression and higher T and N stage as well as increased risk of tumour recurrence after curative resection. On further analysis we identified a cut off score of ≥200 for COX-2 expression, which could serve as a marker of aggressive disease and poor survival. We demonstrated a positive correlation between COX-2 m-RNA expression detected by PCR and COX-2 protein expression detected by immunohistochemistry in patients with oesophageal adenocarcinoma. This further strengthens the validity of our immuno-scoring system and shows a link between genomics and proteomics.

We evaluated COX-2 expression by immunohistochemistry as this is one of the most widely used method that can be used to analyze protein expression in paraffin embedded archival material. Using immunohistochemistry, the signal can be precisely localized to the tumour cell which is not possible when using other techniques like immuno-blot and PCR which can overestimate COX-2 expression by identification of expression within both inflammatory and neoplastic cells. One of the strengths of our work is the specificity of our antibody, as demonstrated by the absence of staining in the antigen adsorption study and in the negative controls. Quantification of protein expression may be difficult when using immunohistochemistry. However, we used an intensity-proportion scoring system which has been extensively used in breast cancer scoring[28]. We found that this scoring system was very easy to use and highly reproducible. The strength of our scoring system was further confirmed by our PCR data, which showed a positive correlation between RT-PCR quantification and immuno-scoring for COX-2.

The literature is fairly divided on the clinicopathologic significance of COX-2 in oesophageal cancer. There are two studies addressing this issue in patients of Japanese origin with squamous cell cancer of oesophagus and have demonstrated a lack of correlation between COX-2 expression and clinicopathologic features of the tumour or overall survival [24,25]. Two additional studies have been performed in patients of western origin with adenocarcinoma of oesophagus and like our study have demonstrated a negative correlation between levels of COX-2 expression and overall survival, although unlike our study no correlation was found between COX-2 expression and clinicopathologic features of the tumour and no attempt was made to correlate the COX-2 protein expression with the COX-2 m-RNA expression[23,26]. Our study substantiates the findings of these previous studies and gives a better insight into the link between the clinico-pathologic features and COX-2 expression in adenocarcinoma of oesophagus. However, another previous study by Lagorce et al [34] found that COX-2 is expressed predominantly in well differentiated adenocarcinoma and did not find any correlation between COX-2 expression and patient survival or any of the clinicopathologic features of the tumour. It is difficult to explain the differences in the findings of this study as compared to ours and the other two studies. However, the study by Lagorce et al included patients with distant metastasis and grouped moderate and well differentiated tumours together producing a slightly skewed distribution in favour of poor differentiation (47 vs 19). Our study excluded patients with distant metastasis and grouped moderate and poorly differentiated tumours together to produce a skew in favour of well differentiation (79 vs 11). These differences in patient selection and study design might explain the differences in results.

The strengths of our study include the large number of enrolled consecutive patients with adenocarcinoma of oesophagus, the use of immunohistochemistry to precisely localise the expression in the neoplastic cells, the use of resection specimens rather then superficial biopsies (as biopsies could underestimate the expression of COX-2 due to its heterogenous nature of expression), the demonstration of a positive link between COX-2 protein (immunohistochemistry) and m-RNA (PCR) expression and the specificity of our antibody as shown by the absence of staining in negative controls and during antigen adsorption study.

Given the current literature and the findings of our study it can be concluded that the prognostic significance of COX-2 overexpression in oesophageal cancer varies with the histological subtype of the tumour, a negative prognostic influence in adenocarcinoma but with no prognostic influence in squamous cell cancer. This raises the possibility that the biological behaviour of the oesophageal cancer might vary with the histological type of the tumour.

It is not clear from our study whether COX-2 plays a primary role in carcinogenesis and progression or is simply a para-phenomenon. However, a primary role of COX-2 is consistent with the epidemiological data linking increased survival and reduced incidence of oesophageal cancer and NSAID use[5]. The precise mechanism for the survival disadvantage observed in patients with high levels of COX-2 is not clear but it is well established that cancer cells expressing high levels of COX-2 have a higher rate of proliferation and a lower rate of apoptosis[29]. Selective inhibition of COX-2 reduces tumourigenesis in different models of carcinogenesis[17,19,30]. Thus, COX-2 has been shown to play a causal role in carcinogenesis. Furthermore, COX-2 expression has been linked to enhanced levels of angiogenesis[31] and expression of metalloproteinases[32] resulting in an increased invasiveness and metastatic potential of tumours. This is further demonstrated by the effect of COX-2 inhibitor in suppressing the haematogenous metastasis of colon cancer in mice[33]. Our study demonstrates an association between high levels of COX-2 expression and higher T-stage, N-stage and an increased risk of tumour recurrence after potentially curative resection. These are all features of a tumour with increased malignant and metastatic potential and this might be one of the mechanisms explaining the link between poor survival and increased COX-2 expression. However, on multivariate analysis we found that the effect of COX-2 on survival was independent of T and N stage. This raises the possibility that there may be other mechanisms by which COX-2 adversely influences survival.

The adverse influence of COX-2 on patient survival has also been reported in other gastrointestinal tumours like the stomach and colon but none of these studies elucidate the mechanisms behind the influence of COX-2 on survival, which still remains a matter for future research.

## Conclusion

This is the first report on higher levels of COX-2 expression being linked to higher T and N stage of tumour, increased risk of tumour recurrence and reduced survival in adenocarcinoma of oesophagus. Although no conclusions with regard to treatment can be drawn from these findings, it does suggest that COX-2 may play a role in increasing the malignant potential of adenocarcinoma of oesophagus. These findings justify the need to initiate future clinical studies exploring the role of COX-2 inhibitors in the treatment of adenocarcinoma of oesophagus with the intention of improving survival. We also believe that estimation of COX-2 expression may serve as a prognostic tool in clinical practice and future interventional research aimed at improving survival in patients with adenocarcinoma of oesophagus.

## Abbreviations

COX: Cyclooxygenase, NSAID: Nonsteroidal anti inflammatory drug

## Competing interests

The author(s) declare that they have no competing interests.

## Authors' contributions

PB: Conception, design, data acquisition, data analysis, data interpretation and drafting manuscript

ACB, RLM, PP: design, data analysis and drafting manuscript

BSFS, PJ, FDN: data acquisition

IAC: data acquisition, drafting manuscript

All authors read and approved the final manuscript

## Pre-publication history

The pre-publication history for this paper can be accessed here:



## Figures and Tables

**Table 1:** Univariate and Multivariate analysis of survival related clinicopathologic parameters by the Cox Proportional Hazards Model

	Univariate			Multivariate		
n = 90	RR	95% CI	p-value	RR	95% CI	p-value
Age	1.01	0.98 – 1.03	0.620	1.02	0.99 – 1.04	0.120
Male gender	0.85	0.40 – 1.78	0.660	1.51	0.69 – 3.28	0.300
Higher T-stage (>T2)	2.79	1.41 – 5.51	0.003	2.25	0.92 – 5.14	0.060
Higher N-stage (>N0)	2.96	1.58 – 5.54	<0.001	2.30	1.25 – 4.23	0.010
Poorly differentiated tumour	1.78	0.94 – 3.36	0.077	1.53	0.73 – 3.20	0.260
High COX-2 score (≥200)	3.53	2.11 – 5.89	<0.001	2.91	1.78 – 4.76	<0.001

**Table 2:** Correlation between COX-2 expression and clinicopathologic characteristics* Log rank test

	Cox-2 score	p value*
T-stage		
T-2 (n = 11)	124.55	
T-3 (n = 79)	199.50	0.008

N-stage		
N-0 (20)	156.00	
N-1 (70)	200.14	0.049

Tumour recurrence		
No (22)	99.54	
Yes (55)	226.72	0.01

Tumour differentiation		
Well (11)	134.50	
Poor/moderate (79)	198.00	0.045
